# LipidLocator: an open source Shiny web application for spatial lipidomics

**DOI:** 10.1093/bioadv/vbag012

**Published:** 2026-01-20

**Authors:** Prateek Arora, Simon Isfort, Nick Kirschke, Mojgan Masoodi, Nadia Mercader

**Affiliations:** Institute of Anatomy, University of Bern, Bern, 3012, Switzerland; Department of BioMedical Research DBMR, University of Bern, Bern, 3008, Switzerland; University Institute for Clinical Chemistry, Inselspital, Bern, 3010, Switzerland; Graduate School of Cell Biology, University of Bern, Bern, 3012, Switzerland; Institute of Anatomy, University of Bern, Bern, 3012, Switzerland; Department of BioMedical Research DBMR, University of Bern, Bern, 3008, Switzerland; Department of BioMedical Research DBMR, University of Bern, Bern, 3008, Switzerland; University Institute for Clinical Chemistry, Inselspital, Bern, 3010, Switzerland; Institute of Anatomy, University of Bern, Bern, 3012, Switzerland; Department of BioMedical Research DBMR, University of Bern, Bern, 3008, Switzerland; Centro Nacional de Investigaciones Cardiovasculares Carlos III, Madrid, 28029, Spain

## Abstract

**Motivation:**

Spatial lipidomics enables the study of how lipids are distributed within tissues, providing insights into tissue structure and function. However, analyzing complex mass spectrometry (MS) imaging (MSI) data remains challenging due to limited tools for high-confidence annotation, especially for integrating MSI, MS, and MS/MS pipelines.

**Results:**

We developed LipidLocator, an open-source, interactive Shiny web application as a unified spatial lipidomics pipeline. LipidLocator integrates MSI data analysis from normalization, spatial clustering, and differential abundance analysis to MS and MS/MS-based lipid annotation. We utilized LipidLocator to analyze DESI-MSI and AP-SMALDI data from adult zebrafish sections, human renal carcinoma, and mouse whole brain sections, to demonstrate its ability to segment distinct anatomical structures and tissue sub-regions and to generate high-confidence lipid profiles using integrated MS and MS/MS annotation. LipidLocator is an end-to-end open-source spatial lipidomics pipeline, facilitating lipid imaging studies in various organisms and covering different lipid detection technologies, providing a valuable and user-friendly resource for investigating lipid metabolism.

**Availability and implementation:**

The LipidLocator application is freely available as a Docker image on Docker Hub at pratarora/lipidlocator. Installation instructions and code are available at https://github.com/MercaderLabAnatomy/LipidLocator.

## 1 Introduction

Lipids play a central role in the functioning of a cell by regulating the cellular structures, energy storage, and signaling. Their spatial distribution contributes to tissue function, and dysregulation can lead to disease (Harayama and Riezman 2018, Melero and Jiménez-Rojo 2023, Seubnooch *et al.* 2024).

Mass spectrometry imaging (MSI) enables in situ visualization of spatially distributed lipids in tissues across diseased or normal conditions (Buchberger *et al.* 2018, Zhang *et al.* 2024). Despite MSI advancements, analytical challenges hinder widespread spatial lipidomics usage. In MSI, each pixel contains MS spectra. Therefore, slides with hundreds of pixels generate high dimensional data. Analyzing these datasets requires robust and user-friendly software for processing, normalization, analysis, and visualization. Current MSI tools often demand high expertise, programming skills, or costly licenses, which limit their accessibility.

A key bottleneck in MSI data analysis is the lack of integrated graphical user interface (GUI) tools, with MS and MS/MS annotations, reducing reproducibility and increasing analysis time. Tools like LipidView enable lipid annotation and quantification but lack MSI support, while Cardinal supports MSI visualization and analysis without lipid identification (Ejsing *et al.* 2006, Bemis *et al.* 2023). Confident lipid identification from MSI data remains challenging. Although resources like LipidBlast, HMDB, and in silico fragmentation aid MS/MS annotation, their integration in user-friendly tools is limited to a few examples including commercially available LipostarMSI (Kind *et al.* 2013, Tortorella *et al.* 2020, Wishart *et al.* 2022). Web apps like ShinyCardinal offer GUI for Cardinal but lack MS/MS-level annotation (Dong and Heinig 2024).

Additionally, large MSI datasets require considerable computing power and expertise (Buchberger *et al.* 2018).

To overcome these limitations, we developed LipidLocator, an interactive Shiny web application providing an end-to-end spatial lipidomics analysis pipeline. LipidLocator integrates Cardinal for MSI and RforMassSpectrometry packages for isotopologue identification, MS, and MS/MS annotation. Its GUI provides access to parameters for normalization and spatial clustering and differential abundance analysis, isotopologue identification and lipid annotations using LIPIDMAPS, LipidBlast, and HMDB databases. To ensure easy installation and reproducibility, LipidLocator is Docker-containerized with the source code freely available.

Here, we describe the implementation of LipidLocator and demonstrate its utility by applying it to a desorption electrospray ionization (DESI)-MSI dataset from adult zebrafish, a key vertebrate preclinical model system. DESI-MSI allows ambient lipid analysis with minimal sample preparation. We show how LipidLocator uses this data to segment organs and generate organ-enriched lipid profiles. We further demonstrate its capabilities on DESI-MSI-Human Renal Cell Carcinoma (RCC) and AP-SMALDI based Mouse brain data, for pairwise tissue comparisons and the analysis of large datasets, showcasing its utility for spatial lipidomics.

## 2 Implementation

**Figure 1:**
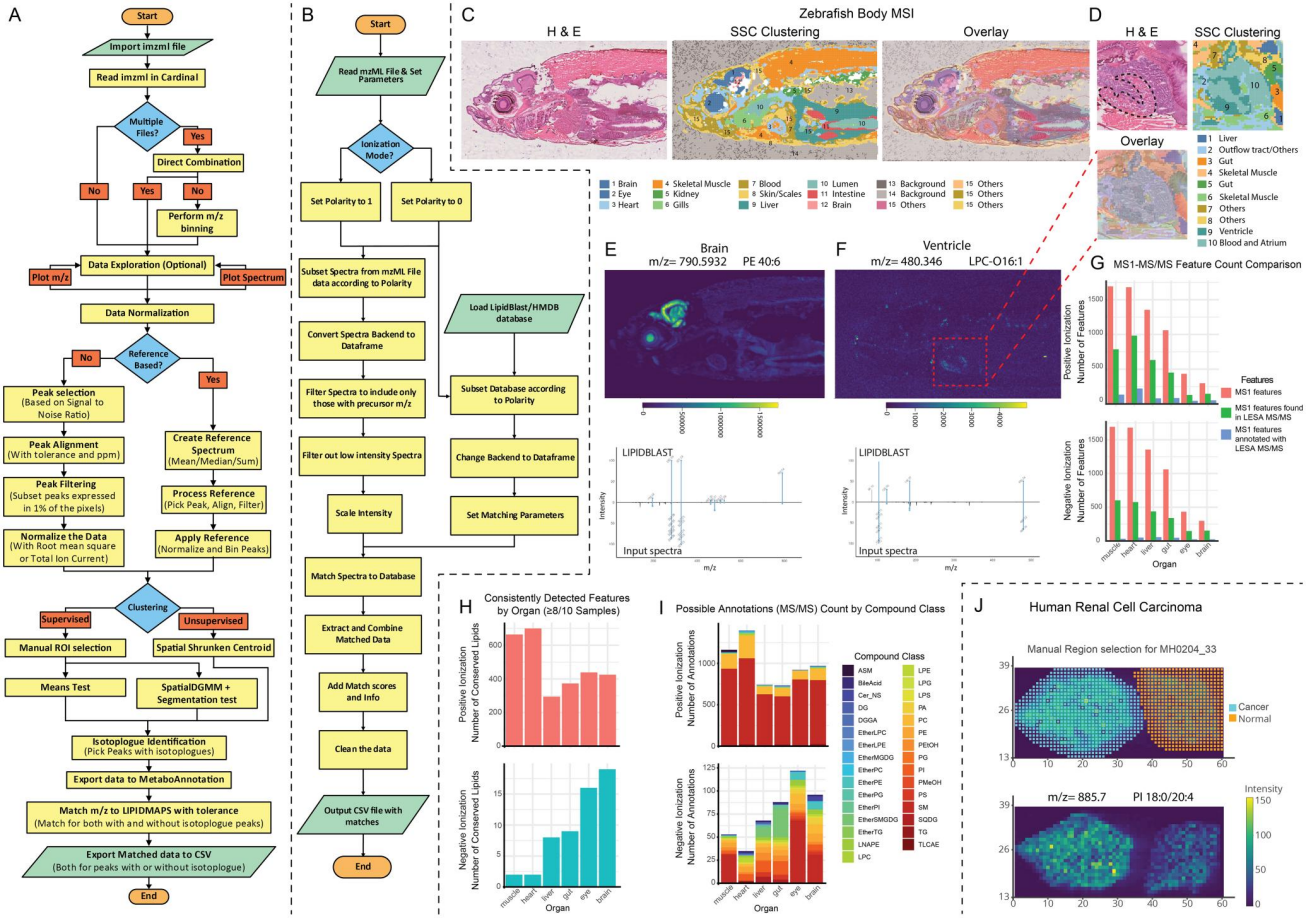
Overview of the LipidLocator pipeline and application. (A) Workflow from DESI-MSI data to MS1 lipid annotations. Data is read using the Cardinal package, followed by peak detection (S/N ratio), alignment (ppm/tolerance), filtering of low-abundance features, normalization, unsupervised spatial clustering, or supervised differential abundance analysis, isotopologue detection, and annotation via LIPIDMAPS. (B) MS/MS workflow: Ionization mode is selected, spectra filtered by precursor *m*/*z* and intensity, scaled for matching, annotated via LipidBlast, and exported after removing duplicates. (C) Zebrafish sagittal section with H&E staining, spatial clusters from SSC, and overlay for organ identification. Colors correspond to organ-specific clusters, with numbered labels. (D) Heart region: H&E, zoomed-in image, SSC clustering, and overlay; ventricle marked by black dotted line. Colors correspond to specific clusters, with numbered labels (E) Brain-enriched PE 40:6 with mirror plot showing LipidBlast match. (F) Ventricle-enriched LPC-O 16:1 with matching spectrum; red box with dotted lines indicates heart region from (D). (G) Bar plot showing number of features at MS1 (DESI, red bars, which are the first bars for each organ), confirmed by MS2 (LESA, green bars, which are the second bars for each organ), and annotated post-MS2 (blue bars, which are the third bars for each organ). (H) Bar plot showing number of features consistently detected in ≥8 of 10 measurements per organ. Ionization modes (positive/negative) refer to MS1 and MS2 acquisition settings. (I) Stacked bar plot showing MS2-annotated features grouped by lipid class. (J) Human Renal Cell Carcinoma samples manually selected with Region of Interest Selector and showing differential enrichment of PI 18:0/20:4 in Cancer versus Normal tissue.

LipidLocator is an interactive R shiny application with a user-friendly interface using shiny.semantic (Methods and Data, available as supplementary data at *Bioinformatics Advances* online). It uses modular UI-server structure and incorporates progress indicators, notifications, error handling and validation steps. The analysis workflow, app walkthrough, and screenshots provide an overview of the app (Fig. 1A and B, Fig. 1, available as supplementary data at *Bioinformatics Advances* online, Movie 1, available as supplementary data at *Bioinformatics Advances* online).

Following the statistical framework for MSI (Bemis *et al.* 2019, 2023), LipidLocator addresses three distinct research questions: (i) Class Discovery or spatial clustering, enabling unsupervised segmentation of tissues based on lipid molecular signatures; (ii) Class Comparison, detecting differentially abundant lipids between conditions or regions; and (iii) Lipid Annotation, assigning chemical identities to spatially resolved features via MS and MS/MS matching.

The application is organized into three main tabs, each corresponding to a major analysis component. In the Data Visualization/Clustering tab, users upload .imzML and .ibd files to the interface. Using Cardinal::readMSIData, data is read into an MSImagingExperiment object supporting multi-tissue experiments for joint processing and visualization of multiple samples. Users can interactively visualize slides per *m*/*z* value, spectra per pixel, and access normalization and clustering controls. Normalization methods including Total Ion Current (TIC) or Root Mean Square (RMS) mitigate analytical variations. Peak processing includes Mean Absolute Deviation, simple, or adaptive methods, followed by peak alignment with user‐defined tolerance and units, and peak filtering using user-defined Signal-to-Noise Ratio. Alongside direct peak processing of individual spectra, LipidLocator offers a reference-based workflow where a spectrum is generated to define a consistent set of *m*/*z* bins, ensuring robust peak alignment across all pixels. For spatial statistical analysis, LipidLocator provides unsupervised clustering and supervised differential abundance testing. For Class Discovery or spatial clustering, LipidLocator uses unsupervised Spatial Shrunken Centroids (SSC) to identify and segment regions with similar lipid profiles. Users can set SSC parameters, including various distance metrics, radius, clusters, and sparsity, and visualize the clustering data in a colored map and table. For Class Comparison or differential abundance analysis, the supervised approach allows users to manually delineate regions of interest (ROIs) based on external knowledge (e.g. H&E staining). LipidLocator identifies differentially abundant features between defined regions using either a means-based statistical test or a more advanced segmentation-based test that uses a spatial Dirichlet Gaussian Mixture Model (spatialDGMM). These methods aggregate pixel-level data into segment-based summaries to account for spatial autocorrelation and biological variation. Critically, this approach ensures that the same features are not used first to define the regions and then again to test for differential abundance between them (Bemis *et al.* 2016, 2019, 2023, Guo *et al.* 2019 ). LipidLocator includes data export and caching using RDS files to reduce re-computation time.

Even without MS/MS data, LipidLocator offers significant utility by providing a user-friendly interface for MSI data processing, spatial clustering, differential abundance testing, isotopologue-based filtering, visualization, and tentative MS1 level lipid annotation.

In the Peak Annotation tab, users can continue with unsupervised clustering, supervised differential analysis, or upload saved data. This module uses the MetaboAnnotation::matchMz function to match detected *m*/*z* features to the LIPIDMAPS database, utilizing user‐specified adducts and tolerances (Rainer *et al.* 2022, Conroy *et al.* 2024, Gatto *et al.* 2025). LipidLocator allows filtering annotations by isotopologue presence to reduce false positives, with output as a downloadable lipid ID table.

MS-based annotations relying solely on accurate mass (*m*/*z*) contain ambiguity from isomers and isobars, when mass differences fall below instrument resolution. This limitation requires the use of tandem mass spectrometry (MS/MS) and spectral matching for more confident lipid identification (Köfeler *et al.* 2021). The MS/MS Analysis tab processes uploaded .mzML files containing MS/MS spectra to perform spectral matching. We utilize MetaboAnnotation::matchSpectra function, to compare experimental query spectra against reference target spectra from LipidBlast or HMDB libraries. For this, we updated CompoundDB (v1.9.2) to parse LipidBlast’s JSON spectra. The MS/MS processing requires the MS precursor within the user-defined ppm/tolerance, to filter out unwanted data. LipidLocator reports the ppm error for mass accuracy check. The matchSpectra function is configured using MatchForwardReverseParam, with peak mapping (MAPFUN, Spectra::joinPeaks) and similarity scoring (MsCoreUtils::ndotproduct), with user-specified ppm/tolerance for spectra matching. The similarity score compares the query spectrum against the target library spectrum. A reverse score does the opposite, effectively considering only the peaks expected in the library spectrum (“right join” logic). The presence ratio quantifies how many library peaks are matched in the query, and high reverse scores and presence ratios indicate strong spectral alignment with the library. Matched peak counts are also reported. LipidLocator displays a mirror plot for each match to directly visualize the spectral peak matches. The final output is a downloadable table of identified lipids and their associated metrics.

LipidLocator is distributed as a Docker image, enabling accessibility and easy installation across operating systems. Furthermore, the containerization and modular design support scalability, allowing LipidLocator’s deployment on various systems from standard desktops to more powerful servers. The underlying Cardinal framework is designed to handle large datasets using out-of-memory data structures, ensuring that LipidLocator remains responsive. To further improve efficiency with large datasets, LipidLocator also supports parallel computation.

## 3 Results and discussion

Firstly, we utilized LipidLocator for spatial lipidomics of adult zebrafish sagittal sections. We used data from DESI‐MSI and targeted Liquid Extraction Surface Analysis (LESA)‐MS/MS in positive and negative ionization modes, using both MS and MS/MS level data (Methods and Data, available as supplementary data at *Bioinformatics Advances* online).

LipidLocator clustered DESI-MSI data into lipid-enriched regions corresponding to anatomical organs including brain, liver, heart, skeletal muscle, and gut (Fig. 1C). We confirmed organ identification by overlaying clusters on Hematoxylin and Eosin (H&E)-stained slides manually in Adobe Photoshop and reducing opacity of both images for better visualization. Subsequent isotopologue removal and database matching based on mass accuracy enabled tentative identification of numerous lipid features. We demonstrated high-resolution clustering of DESI-MSI data by subsetting an area around a specific organ combined with a lower clustering radius to achieve tissue-level lipid signatures. This was exemplified by the heart and the detection of a specific cluster for the cardiac ventricle (Fig. 1D). Features consistently detected in ≥4 of 5 samples per organ cluster were selected for LESA-MS/MS confirmation (Table 1, available as supplementary data at *Bioinformatics Advances* online, Methods and Data, available as supplementary data at *Bioinformatics Advances* online).

The acquired MS/MS spectra (.mzML files) were processed in LipidLocator for annotation. Spectral matches against LipidBlast provided high-confidence annotations for the lipid features previously identified in each organ. In the brain, LipidLocator annotated PE 40:6 (*m*/*z* 790.5380), with strong evidence: reverse score 0.829, presence ratio 1.0, 8 matched peaks, and 1.45 ppm error. The interactive mirror plot in LipidLocator provided clear visual confirmation of this match (Fig. 1E). PE 40:6 has previously been reported to be dysregulated in brains of Alzheimer’s disease patients (Mendis et al., 2016). Similarly, other enrichments were identified in eye, liver, gut, and cardiac ventricle subclusters among others (Fig. 1F, Fig. 2, available as supplementary data at *Bioinformatics Advances* online, Table 1, available as supplementary data at *Bioinformatics Advances* online, Methods and Data, available as supplementary data at *Bioinformatics Advances* online).

We evaluated LipidLocator’s performance in a multistage workflow (Fig. 1G and H). We counted the features that were consistently present in ≥4 out of 5 replicates (red bars) in DESI-MSI, features detected post- LESA-MS/MS (green bars) and features annotated using LipidBlast (blue bars). Annotated features (by MS/MS spectral match) relative to MS/MS-targeted features varied across tissues: in positive mode: muscle 134/786 (17.0%), heart 222/983 (22.6%), liver 80/632 (12.7%), gut 85/451 (18.8%), eye 44/129 (34.1%), brain 52/146 (35.6%); in negative mode: muscle 32/602 (5.3%), heart 50/576 (8.7%), liver 56/438 (12.8%), gut 47/340 (13.8%), eye 25/147 (17.0%), brain 25/153 (16.3%).

Overall, LipidLocator identified lipid features consistently present in ≥8 of 10 samples per organ (2 spots per fish section, 5 different fish sections) (Fig. 1H). In positive mode, this included 425 features in brain, 439 in eye, 295 in liver, 373 in gut, 700 in heart, and 664 in muscle. In negative mode, 19 features in brain, 16 in eye, 8 in liver, 9 in gut, and 2 each in heart, and muscle features were found (Table 2, available as supplementary data at *Bioinformatics Advances* online). The classes of lipids of the possible annotations were plotted for all five organs to demonstrate the lipid classes identified by LipidLocator (Fig. 1I).

We further demonstrate LipidLocator’s applicability on two additional datasets. We reanalyzed a human RCC dataset that was previously utilized as an example dataset in Cardinal, and processed all eight samples together in LipidLocator (Dill *et al.* 2010). We performed clustering and identified differential features by defining manual regions followed by meansTest for cancer and normal tissues per slide. We then confirmed the presence of previously identified lipids with the Peak Annotation module of LipidLocator including PI 18:0/20:4 (*m*/*z* 885.467) (Fig. 1J, Fig. 3A, available as supplementary data at *Bioinformatics Advances* online), PS 18:0/18:1 (*m*/*z* 788.544), PG(18:1/18:1) (*m*/*z* 774.541), PS(18:0/20:4) (*m*/*z* 811.536) and others (Table 3, available as supplementary data at *Bioinformatics Advances* online). To demonstrate the scalability, we analyzed a whole mouse brain slide with 148 406 pixels (Bassini *et al.* 2025). We performed the SSC clustering and found clusters for the regions of cortex, cerebral cortex, midbrain and others. MS1 Peak annotations showed previously reported PC 40:1 (*m*/*z* 844.8604) (Fig. 3B, available as supplementary data at *Bioinformatics Advances* online), PC 38:3 (*m*/*z* 811.6091), PE 34:1 (*m*/*z* 717.5309) and PE 44:8 (*m*/*z* 843.5778) (Table 4, available as supplementary data at *Bioinformatics Advances* online) amongst others confirming the reproducibility of the pipeline across species and tissues.

In summary, we developed LipidLocator, an open-source application that uniquely integrates MSI spatial analysis processing with MS and MS/MS annotation into a single reproducible pipeline. LipidLocator supports multi-tissue experimental designs, enabling clustering and differential abundance analysis that is applicable across various biological systems. It represents a powerful resource for the spatial omics community and sets the stage for future enhancements, such as pathway analysis tools.

## Supplementary Material

vbag012_Supplementary_Data

## Data Availability

The LipidLocator application is freely available as a Docker image on Docker Hub at pratarora/lipidlocator at https://hub.docker.com/r/pratarora/lipidlocator. Installation instructions and code are available at https://github.com/MercaderLabAnatomy/LipidLocator. The data used for the publication are available under DOIs 10.5281/zenodo.15473321, 10.5281/zenodo.15473636, 10.5281/zenodo.15473195, 10.5281/zenodo.15112789.
