# Supplementary material for: LipidLocator: an open source Shiny web application for spatial lipidomics
Source: Bioinform Adv. 2026 Jan 20;6(1):vbag012. doi: 10.1093/bioadv/vbag012 (PMC12883462; doi:10.1093/bioadv/vbag012)
Supplement: vbag012_Supplementary_Data [file vbag012_supplementary_data.zip › Supplementary Methods and Data_Final.docx]

# **Supplementary Results**

# **Lipid Annotations Across Zebrafish Organs**

LipidLocator annotated lipids across different organs and across both positive and negative ionizations. In positive ionization, we observed enrichment in sphingomyelin (SM) and phosphatidylcholine (PC) classes in the brain. We identified species such as SM (d41:1) and SM (d32:1) consistent with previous MSI findings in the zebrafish brain and spinal cord (Sohn et al., 2024; Sun et al., 2023). We also detected enrichments of lysophosphatidylcholines (LPC), diglycerides (DG), and ether‐linked PCs (EtherPC) (Supplementary Table S2). Negative mode analysis using LipidLocator indicated enrichments of Ceramide (Cer), phosphatidylethanolamine (PE), Phosphatidylinositol (PI), and PC classes in the brain cluster, consistent with previous reports (Liang et al., 2021; Sun et al., 2023). Additionally, LipidLocator also annotated other enriched lipids in this mode, such as lysophosphatidylglycerols (LPG) and lysophosphatidylinositol acyl‐phosphatidylethanolamines (LNAPE) (Supplementary Table S2). The eye lipid profile showed similar lipid classes as in brain. In positive mode we observed PCs, LPCs, Dg and EtherPC, such as PC 34:1 (m/z 760.5941) (Supplementary Figure 2A) (Supplementary Table S2) In negative mode we observed Cer, PE, PI, and PC, LNAPE and LPG classes showing enrichment in both brain and eye (Supplementary Table S2) (Liang et al., 2021; Sun et al., 2023). In liver, we observed LPE, LPG, PC and PI lipid classes, such as PI 38:5 (m/z 883.5331) as predominant features in negative mode (Supplementary Figure 2B) and SM and PC classes in positive mode (Supplementary Table S2) (Sun et al., 2023). In the intestine, PCs and PIs were prominent in both ionization modes (Sun et al., 2023), We also observed various PC species including EtherPC, LPC, DG, and TG (positive mode), and LPE, LPG, and PI species like PI 36:3 (m/z 859.5331) in negative mode (Supplementary Figure 2C). Skeletal muscle tissue showed diverse PC/SM annotations in positive mode as previously observed (Sun et al., 2023). In the negative ionization mode, the muscle showed enrichment of PIs and other PC classes. The heart subclusters showed a diverse range of PC and SM species in positive mode, as previously reported (Sun et al., 2023) and enrichments such as PI, PC and LysoPhosphatidylEthanolamines (LPE) classes including LPE 16:0 and LPE 18:1 in negative mode (Supplementary Table S2). Furthermore, subclustering also detected the enrichment of a subset of these species, including LPC-O 16:1 (m/z 480.346) (Figure 1F), in the cardiac ventricle.

Thus, LipidLocator overall allowed the confirmation of many known lipids previously detected in other species (Sun et al., 2023; Sohn et al., 2024; Liang et al., 2021). Some of them had been previously also related to disease. For example, PE (40:6) found in our study in the zebrafish brain has been previously reported in mouse and human brains to be dysregulated in hippocampi in Huntington’s disease transgenic mice or Alzheimer’s disease patients, (Mendis et al., 2016; Farzana et al., 2023). PC 34:1 detected in the zebrafish eye also showed cross-species conservation and has been previously reported in human and salamander retina (Roy et al., 2011; Zemski Berry et al., 2014). We found PI 38:5 enriched in the liver, which has previously been reported in mouse liver mid-zone and associated with fatty liver in children. (Seubnooch et al., 2023; Mann et al., 2022; Seubnooch et al., 2024). In the gut, we found PI 36:3 enrichment, which has been shown to be present on human villi tips and associated with Acute Radiation Syndrome of the gastrointestinal tract (Carter et al., 2019). Similarly, skeletal muscle tissue showed conservation of PI(38:4) across humans and zebrafish (Bacle et al., 2020). In the heart, sub-clustering identified the unreported enrichment of LPC-O (16:1) in the ventricle. Thus, we found representation of lipids in brain, eye, liver, intestine, and skeletal muscles that are also observed in humans or mice.

We observed that due to spectral matching limitations, such as inability to resolve double bond positions or stereospecificity, some matches yielded multiple possible annotations. This was particularly prominent for SMs, potentially leading to class overrepresentation (Figure 1I). Furthermore, we observed that annotations were more frequent in positive than negative ionization mode, likely reflecting limited compatibility of zebrafish lipids with human-centric databases. Some lipids may be zebrafish-specific or uncharacterized, requiring further structural validation. Thus, the relatively modest number of annotated features may reflect species-specific biological differences and reference databases limitations rather than limitations of the pipeline itself.

Overall, these examples illustrate LipidLocator's ability to identify biologically relevant lipids within their spatial context.

# **Supplementary Methods**

# **Animal husbandry**

Zebrafish (*Danio rerio*) of the wildtype strain AB were held at the fish facility of the Institute of Anatomy (National License number 35) and used under license BE87/2023 approved by the authorities of the Canton of Bern. Housing conditions for zebrafish were 28°C temperature, 650–700 μS/cm conductivity, and pH 7.5; 10% water exchange per day and lighting conditions were 14:10 h (light: dark). Animals were grown to adulthood and euthanized by anaesthesia overdose using 0.16% Tricaine (Sigma, St Louis, MO, USA) followed by deep freezing. Experiments were performed with adult zebrafish aged nine months. Experiments were performed with five male adult zebrafish for MS1 and five male adult zebrafish for MS/MS confirmation.

# **Preparation of zebrafish slides**

5% Carboxymethylcellulose (CMC) solution was dissolved without gelatin and kept in a boiling setup until usage as previously described (Nelson et al., 2013). Post euthanasia, the fish were dried and placed inside a plastic mold on top of a small layer of cooled CMC medium. After placing the fish in an adequate horizontal position, a cooled-down portion of CMC medium was poured into the mold avoiding air bubbles, until the whole fish was covered. A beaker filled with absolute Ethanol (EtOH) was placed on dry ice and a few bricks of dry ice were placed inside that beaker to further cool down the EtOH. The mold was placed on top of a floating aluminium foil boat to avoid sinking and therefore rapid cooling of the sample. The mold was kept in the pre-cooled EtOH until the whole medium was frozen. Samples were then stored at -80°C or used directly for cryosectioning.

The cryostat (Thermo Scientific CryoStar NX50) was cooled to -20°C, and the 16 µm-thick sections were cut using MX35 Premier cutting knife. A special brush set exclusively for metabolomics sectioning was used to avoid contamination. All the tools for cutting were placed inside the cryostat for at least 30 min before usage. Excess medium around the fish sample was removed using razor blades. Milli-Q water was used to place the sample on a specimen disk. The specimen disk was positioned such that the fish’s ventral side met the cutting knife first. Upon reaching the swim bladder, CMC medium was filled in with a syringe and cooled down for at least 15 min before proceeding with the sectioning. Some sections were stained with toluidine blue to confirm the presence of all organs. The sections were collected on slides, air-dried for a maximum of 5 min, and placed inside a Styrofoam box with dry ice and stored in -80°C.

The H&E staining was performed as previously described (Stutts et al., 2020). In brief, the slides were fixed in 10% Formalin for 1 h, rinsed in deionized water, washed in a descending EtOH series for 30 s each (95% EtOH followed by 70% EtOH), stained for 45 s in Haematoxylin (Harris), followed by an ascending Ethanol series for 30 s each (70% EtOH followed by 95% EtOH), stained for 1 min in Eosin, rinsed in high percentage EtOH for 30 s each (90% EtOH followed by 100% EtOH), soaked in Xylene for 2 min and finally covered with a cover slip, preventing bubbles. The H&E stained images were manually overlaid with SSC clustering in Adobe Photoshop with reduced opacity of both the images to confirm the organ identification.

# **Software and Implementation**

The spatial lipidomics data analysis was performed in LipidLocator, using a developed interactive Shiny application in R (version 4.3.2). The application is built using the ‘shiny’, ‘shiny.semantic’, ‘shinycssloaders’ packages for the user interface and leverages several bioinformatics R packages for data processing and analysis, including ‘Cardinal’, ‘Spectra’, ‘MetaboAnnotation’, ‘MetaboCoreUtils’, ‘CompoundDb’, ‘ChemmineR’, ‘mzR’, and ‘MsCoreUtils’. Data manipulation and visualization were performed using ‘dplyr’, ‘data.table’, ‘dtplyr’, ‘ggplot2’, ‘DT’, ‘plotly’ and ‘paletteer’. Parallel processing was implemented using ‘BiocParallel’ to improve computational efficiency (Chang et al., 2023; Stachura et al., 2021; Bemis et al., 2023; Rainer et al., 2022; Martens et al., 2010; Cao et al., 2008; Wickham et al., 2018; Dowle et al., 2018; Wickham et al., 2019; Xie et al., 2023; Hvitfeldt, 2021; Morgan et al., 2023; Sali and Attali, 2020; Sievert, 2020).

The application was structured in a modular fashion, with distinct modules for each step of the analysis workflow. These modules are interconnected within a user-friendly tabset interface, guiding users through data upload, exploration, normalization, clustering, peak annotation, and MS/MS analysis. LipidLocator was designed to be deployed on a server environment via Shiny Server or similar platforms.

# **Data Input and Preprocessing**

LipidLocator accepts spatial MSI data in the .imzML format along with its corresponding .ibd file. Upon uploading the data via the "Data Visualization/Clustering" tab, the application utilizes the ‘readMSIData’ function from the ‘Cardinal’ package to read the .imzML and .ibd files into an ‘MSIDataSet’ object.

# **Data Exploration and Visualization**

The "Data Visualization/Clustering" tab provides tools for initial data exploration. Users can visualize m/z intensity distributions across the spatial domain using image plots, allowing for the inspection of spatial patterns for specific m/z values. The application also enables the visualization of mass spectra for individual pixels, selected by their x and y coordinates, facilitating the assessment of spectral quality and feature distribution across different spatial locations. For datasets containing multiple runs or slides, key visualizations such as ion heatmaps, pixel spectra, and SSC clustering results are presented in a faceted grid, facilitating direct side-by-side comparison. All plots are rendered interactively using the Plotly library, allowing users to zoom, pan, and inspect individual data points that can be exported as high-quality SVG files for publication.

# **Data Normalization and Feature Extraction**

For data normalization and feature extraction, the "Data Visualization/Clustering" tab offers a range of options. Users can select from different normalization methods, including Root Mean Square (RMS) and Total Ion Current (TIC) normalization, implemented using the ‘normalize’ function from the ‘Cardinal’ package. Peak picking is performed using methods such as Mean Absolute Deviation (MAD), Simple (based on standard deviation), and Adaptive noise estimation, leveraging the ‘peakPick’ function in ‘Cardinal’. The signal-to-noise ratio (SNR) threshold for peak picking is a user-adjustable parameter. Alongside direct peak processing of individual spectra, LipidLocator offers a reference-based workflow where a spectrum is generated to define a consistent set of m/z bins, ensuring robust peak alignment across all pixels. For peak alignment across spectra, the application utilizes the ‘peakAlign’ function within ‘Cardinal’, allowing users to define a tolerance value and units (m/z or ppm). Extracted features, represented as m/z values and their corresponding intensities, are stored within the processed ‘MSIExperiment’ object.

At the completion of the normalization and feature extraction step, the application provides users with the option to download the normalized data object as a CSV file. This functionality allows users to inspect and utilize the feature list independently.

# **Spatial Clustering**

Spatial segmentation of the MSI data is performed using the Spatial Shrunken Centroids (SSC) clustering algorithm, as implemented in the ‘spatialShrunkenCentroids’ function of the ‘Cardinal’ package. Users can select between Gaussian and Adaptive spatially-aware weight methods for the SSC algorithm. The distance metric for clustering can be chosen from Radial, Manhattan, Minkowski, and Chebyshev distances. Key parameters for SSC clustering, including the spatial neighborhood radius (r), the maximum number of clusters (k), and the sparsity threshold (s), are user-adjustable, allowing for optimization of cluster segmentation. The clustering results are visualized as spatial segmentation maps overlaid on the tissue image.

Upon completion of spatial clustering, the application offers the capability to download the clustered ‘MSIExperiment’ object as an RDS file, preserving the clustering model and segmentation. Furthermore, a table of clustered features, including feature statistics and cluster assignments, can be downloaded as a CSV file. This allows users to examine the features contributing to each spatial cluster in detail.

# **Manual Region Selection and Differential Analysis**

As an alternative to unsupervised clustering, LipidLocator includes a module for manual Region of Interest (ROI) selection and supervised differential analysis. Users can interactively define ROIs by drawing polygons directly onto the ion images using the plotly interface. A custom point-in-polygon algorithm maps the user-defined vertices to the underlying pixel coordinates, ensuring precise capture of anatomical structures. The module supports multi-run datasets, allowing users to define and merge regions across multiple tissue slides while maintaining sample identity.

Once regions are defined (e.g., Region 1 vs. Region 2), the application extracts features using one of two statistical approaches provided by the Cardinal package. The first approach is a Segmentation-based test (spatialDGMM) that utilizes Spatially-aware Dirichlet Gaussian Mixture Models (spatialDGMM). To optimize performance, features are first filtered based on a user-defined variance quantile threshold to remove low-variance noise. The application then performs spatial segmentation within the cropped ROIs followed by a segmentationTest to identify features that significantly distinguish the defined regions. The second approach is a Means-based test. This method utilizes the meansTest function, which compares the mean intensities of features across the defined regions using a linear model, identifying lipids that are differentially abundant between the selected ROIs. Results from the manual analysis, including feature statistics, p-values, and fold changes, are generated as downloadable CSV files, and the processed ROI data objects can be saved for reproducibility.

# **Peak Annotation and Matching**

The "Peak Annotation" tab facilitates the annotation of detected peaks against the LIPIDMAPS database. Users can upload previously clustered data or utilize the clustered data directly from the "Data Visualization/Clustering" tab. Adduct selection is a critical step, with the application allowing users to specify positive, negative, or both ionization modes and to select specific adduct ions from predefined lists relevant to lipidomics analysis. Peak matching is performed using the ‘matchMz’ function from the ‘MetaboAnnotation’ package against a local ‘CompDb’ database derived from LIPIDMAPS. Users can define the mass tolerance in m/z or ppm for peak matching. Due to multiple possible annotations for each m/z value the table increases in size exponentially. To handle this massive table data frame, we employ ‘dtplyr’ which runs on data.table backend to handle huge data frames efficient and fast manner. The application provides downloadable tables of matched peaks and isotopologue-matched peaks, including relevant compound annotations from the LIPIDMAPS database (Conroy et al., 2024).

The peak annotation module generates and allows for the download of two distinct tables in CSV format: "Matched Peaks" and "Isotopologue Matched Peaks". The "Matched Peaks" table contains a list of peaks from the clustered MSI data that have been matched to entries in the LIPIDMAPS database based on m/z proximity and selected adducts. This table includes key information such as target compound IDs, names, formulas, exact masses, adducts, and the m/z values of the matched peaks from the experimental data. The "Isotopologue Matched Peaks" table provides similar information but specifically for peaks identified as isotopologues, offering insights into isotopic distributions of identified lipids. Both tables also include matching scores and error metrics (ppm), facilitating the assessment of match quality. These downloadable tables serve as crucial outputs for identifying potential lipids within the spatial MSI dataset.

# **MS/MS Analysis and Lipid Identification**

For in-depth lipid identification, the "MS/MS Analysis" tab enables spectral matching of MS/MS data against lipid databases. Users upload .mzML files containing MS/MS spectra. The application supports both positive and negative ionization modes and allows users to set mass tolerance and intensity thresholds for spectral processing. Spectral matching is performed using functions from the ‘Spectra’ and ‘MetaboAnnotation’ packages against databases such as LIPIDBLAST and HMDB, which are integrated as ‘CompDb’ objects (Kind et al., 2013; Wishart et al., 2022). To properly process the in silico–generated spectral libraries of LipidBlast, we updated the CompoundDB code (version 1.9.2) to parse spectrum information from the JSON file format. We have enabled the requirement for MS1 precursor which performs an initial filtering of the data, only comparing query and target spectra if their precursor m/z values match within the defined ppm/tolerance. It then scales the intensities to help match with the databases. LipidLocator also reports the calculated ppm error between the matched query and target precursors, serving as an essential mass accuracy check. The matchSpectra function is configured via the MatchForwardReverseParam object to provide multiple layers of evidence for the matching results. We implemented the peak mapping (MAPFUN, Spectra::joinPeaks) and similarity calculation (MsCoreUtils::ndotproduct) functions, with user-specified ppm and tolerance for matching fragment ions in the primary spectral comparison. The similarity score is calculated by comparing the query spectrum against the target library spectrum and reflects the overall alignment between the two spectra. Further, MatchForwardReverseParam allows reverse score calculation, which is calculated by comparing the target (library) spectrum back against the query spectrum, effectively considering only the peaks expected in the library spectrum ("right join" logic). A similar approach is also used by other tools like MS-DIAL (Tsugawa et al., 2015). A high reverse score indicates that the query spectrum provides good evidence for the peaks expected in the library spectrum, helping to penalize mismatches due to noise or unassigned peaks in the query. A presence ratio is calculated alongside the reverse score, this metric quantifies the proportion of the target (library) spectrum's peaks that were successfully matched (found above threshold) in the query spectrum. A high presence ratio suggests that a significant portion of the expected fragmentation pattern was experimentally observed, bolstering confidence. We also include matched peaks count to report the absolute number of fragment peaks found to match between the query and target spectra above the defined thresholds. The thresholds are applied internally on the minimum score requirements before results are returned, ensuring only reasonably good matches are considered for further evaluation. LipidLocator presents matches passing these initial thresholds. Results are presented in interactive tables and mirror plots, facilitating visual comparison of experimental and database spectra for confident lipid identification.

A key feature of the MS/MS analysis module is the interactive generation of mirror plots. Upon selecting a row in the results table, the application dynamically creates a mirror plot using the ‘plotSpectraMirror_ggplot’ function. These plots visually juxtapose the experimental MS/MS spectrum from the uploaded .mzML file (plotted downwards) against the corresponding database MS/MS spectrum from LIPIDBLAST or HMDB (plotted upwards). The plots facilitate visual validation of spectral matches, allowing users to assess the similarity in fragment ion patterns and intensities between experimental and database spectra. The plots are generated with user-defined ppm tolerance for peak matching and include labels for prominent peaks to aid in visual interpretation.

Following MS/MS spectral matching, the application provides a downloadable CSV file containing the comprehensive lipid analysis results. This table includes detailed information for each identified lipid, such as compound names, formulas, database identifiers, spectral matching scores (forward and reverse scores, matched peak counts, presence ratio), mass accuracy (ppm error), precursor m/z values from both query and target spectra, and relevant metadata from the LIPIDBLAST or HMDB database (e.g., compound class, InChI keys, SMILES notations). This downloadable table serves as the final output of the MS/MS analysis workflow, providing a structured and comprehensive list of identified lipids.

# **Legends for Supplementary data**

**Supplementary Figure 1:** Overview of LipidLocator App. (A) Screenshots showing the module for Data Visualization and Clustering. This module allows data upload, and visualization of intensities for a particular m/z or the whole spectra for a pixel. In the next step it allows for Normalization followed by a clustering module. (B) Peak Annotation. Here, the previously clustered data can be continued or saved data can be uploaded. This is followed by Adduct selection and Peak Matching using m/z and LIPIDMAPS. Users can then download the annotations of peaks with isotopologues or all the peaks. (C) MS/MS module. Here, users can upload the spectra files and select the parameters for annotation. Clicking on one of the matches shows the spectra mirror plot showing the how many peaks matched with the database. Red boxes indicate the key components of the interface.

**Supplementary Figure 2:** Organ enriched lipids with their respective mirror plots showing matching spectra with the LipidBlast database in eye- PC 34:1 (A), liver- PI 38:5 (B), and intestine- PI 36:3 (C). The dashed lines mark the regions of the organs mentioned.

**Supplementary Figure 3:** Application of LipidLocator on Human Renal Cell Carcinoma and Mouse Brain. (A) shows differential enrichment of PI 18:0/20:4 in Cancer vs Normal across eight Human Renal Cell Carcinoma samples processed together. (B) Female Mouse Brain (217D as derived in Metaspace) section showing Spatial Shrunken Centroid clustering and enrichment of PC 40:1 in the cortex marked by cluster number 6, and 11.

**Supplementary Movie 1.** Walkthrough of LipidLocator: Movie showing the walkthrough of the app showcasing all features of the app.

**Supplementary Table S1.** MS1 Annotations after clustering: Table showing lipids that are common across four out of five zebrafish in positive or negative ionization mode as matched using LIPIDMAPS. The table contains the m/z, the possible adducts lipid class and other details from LIPIDMAPS.

**Supplementary Table S2.** Annotation after LESA MS: Tables showing the annotated lipids that were observed in at least eight out of ten measurements in each organ in each positive and negative ionization mode. The tabs give list and details for each of the lipid along with its spectra match scores and other lipid details.

**Supplementary Table S3.** MS1 Annotation of Human Renal Cell Carcinoma. Table shows summarized lists of annotated lipids from all eight RCC samples in negative ionization mode as matched using LIPIDMAPS. The table contains the m/z, the possible adducts lipid class and other details from LIPIDMAPS.

**Supplementary Table S4.** MS1 Annotation of Female Mouse whole brain section.: Table shows summarized lists of annotated lipids from female mouse brain 217D (as derived in Metaspace) in positive ionization mode as matched using LIPIDMAPS. The table contains the m/z, the possible adducts lipid class and other details from LIPIDMAPS.

# **References**

Bacle A, *et al.* A comprehensive study of phospholipid fatty acid rearrangements in metabolic syndrome: correlations with organ dysfunction. *Dis Model Mech* 2020;13:dmm046185.

Bemis KA, *et al.* Cardinal v.3: a versatile open-source software for mass spectrometry imaging analysis. *Nat Methods* 2023;20:1883–6.

Brown DG, *et al.* Metabolomics and metabolic pathway networks from human colorectal cancers, adjacent mucosa, and stool. *Cancer Metab* 2016;4:11.

Cao Y, *et al.* ChemmineR: a compound mining framework for R. *Bioinformatics* 2008;24:1733–4.

Carter CL, *et al.* Characterizing the natural history of acute radiation syndrome of the gastrointestinal tract: combining high mass and spatial resolution using MALDI-FTICR-MSI. *Health Phys* 2019;116:454–72.

Chang W, *et al.* shiny: Web Application Framework for R. 2023. R package.

Conroy MJ, *et al.* LIPID MAPS: update to databases and tools for the lipidomics community. *Nucleic Acids Res* 2024;52:D1677–82.

Dowle M, *et al.* data.table: Extension of ‘data.frame’. R package version 1.10.4-3, 2018.

Farzana F, *et al.* Longitudinal spatial mapping of lipid metabolites reveals pre-symptomatic changes in the hippocampi of Huntington’s disease transgenic mice. *Neurobiol Dis* 2023;176:105933.

Hvitfeldt E. paletteer: Comprehensive Collection of Color Palettes. 2021. R package.

Kind T, *et al.* LipidBlast in silico tandem mass spectrometry database for lipid identification. *Nat Methods* 2013;10:755–8.

Lee T, *et al.* Oral versus intravenous iron replacement therapy distinctly alters the gut microbiota and metabolome in patients with IBD. *Gut* 2017;66:863–71.

Liang X, *et al.* Three-dimensional imaging of whole-body zebrafish revealed lipid disorders associated with Niemann–Pick disease type C1. *Anal Chem* 2021;93:8178–87.

Mann JP, *et al.* Comparison of the lipidomic signature of fatty liver in children and adults. *J Pediatr Gastroenterol Nutr* 2022;74:734–41.

Martens L, *et al.* mzML - a community standard for mass spectrometry data. *Mol Cell Proteomics* 2010;9:2519-22.

Mendis LHS, *et al.* Hippocampal lipid differences in Alzheimer’s disease: a human brain study using matrix‐assisted laser desorption/ionization‐imaging mass spectrometry. *Brain Behav* 2016;6:e00507.

Morgan M, *et al.* BiocParallel: Bioconductor facilities for parallel evaluation. 2023. R package.

Nelson KA, *et al.* Optimization of whole-body zebrafish sectioning methods for mass spectrometry imaging. *J Biomol Tech* 2013;24:119–27.

Rainer J, *et al.* A modular and expandable ecosystem for metabolomics data annotation in R. *Metabolites* 2022;12:173.

Roy MC, *et al.* Salamander retina phospholipids and their localization by MALDI imaging mass spectrometry at cellular size resolution. *J Lipid Res* 2011;52:463–70.

Sali A, Attali D. shinycssloaders: Add Loading Animations to a ‘shiny’ Output While It’s Recalculating. 2020. R package.

Seubnooch P, *et al.* Characterisation of hepatic lipid signature distributed across the liver zonation using mass spectrometry imaging. *JHEP Rep* 2023;5:100725.

Seubnooch P, *et al.* Spatial lipidomics reveals zone-specific hepatic lipid alteration and remodeling in metabolic dysfunction-associated steatohepatitis. *J Lipid Res* 2024;65:100599.

Sievert C. Interactive Web-Based Data Visualization with R, plotly, and shiny. *Chapman and Hall/CRC*, 2020.

Sohn AL, *et al.* Oversampling for enhanced spatial resolution of zebrafish by top-hat IR-MALDESI-MSI. *J Am Soc Mass Spectrom* 2024;35:1959–68.

Stachura F, *et al.* shiny.semantic: Semantic UI Support for Shiny. 2021. R package.

Stutts WL, *et al.* Methods for cryosectioning and mass spectrometry imaging of whole-body zebrafish. *J Am Soc Mass Spectrom* 2020;31:768–72.

Sun Y, *et al.* Spatially resolved metabolomics method for mapping the global molecular landscape of whole-body zebrafish (Danio rerio) using ambient mass spectrometry imaging. *Anal Chem* 2023;95:9164–72.

Tsugawa H, *et al.* MS-DIAL: data-independent MS/MS deconvolution for comprehensive metabolome analysis. *Nat Methods* 2015;12:523–6.

Wickham H, *et al.* dplyr: A Grammar of Data Manipulation. R package version 0.7.6, 2018.

Wickham H, *et al.* Welcome to the Tidyverse. *J Open Source Softw* 2019;4:1686.

Wishart DS, *et al.* HMDB 5.0: the human metabolome database for 2022. *Nucleic Acids Res* 2022;50:D622–31.

Xie Y, *et al.* DT: A Wrapper of the JavaScript Library ‘DataTables’. 2023. R package.

Zemski Berry KA, *et al.* Spatial organization of lipids in the human retina and optic nerve by MALDI imaging mass spectrometry. *J Lipid Res* 2014;55:504–15.
