# Supplementary figures and images for: LipidLocator: an open source Shiny web application for spatial lipidomics
Source: Bioinform Adv. 2026 Jan 20;6(1):vbag012. doi: 10.1093/bioadv/vbag012 (PMC12883462; doi:10.1093/bioadv/vbag012)

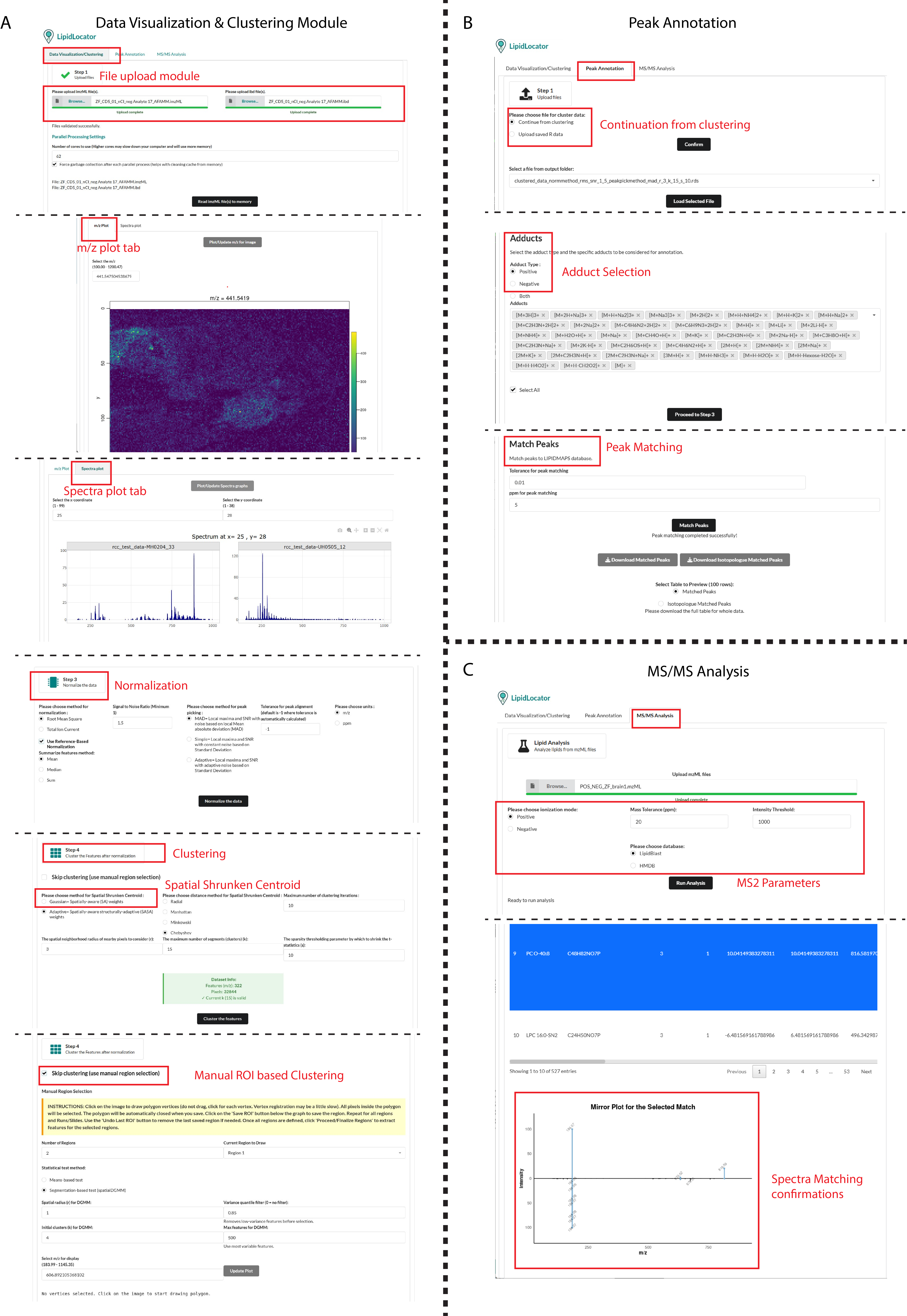

Supplement: vbag012_Supplementary_Data [file vbag012_supplementary_data.zip › Supplementary Fig 1.tif]

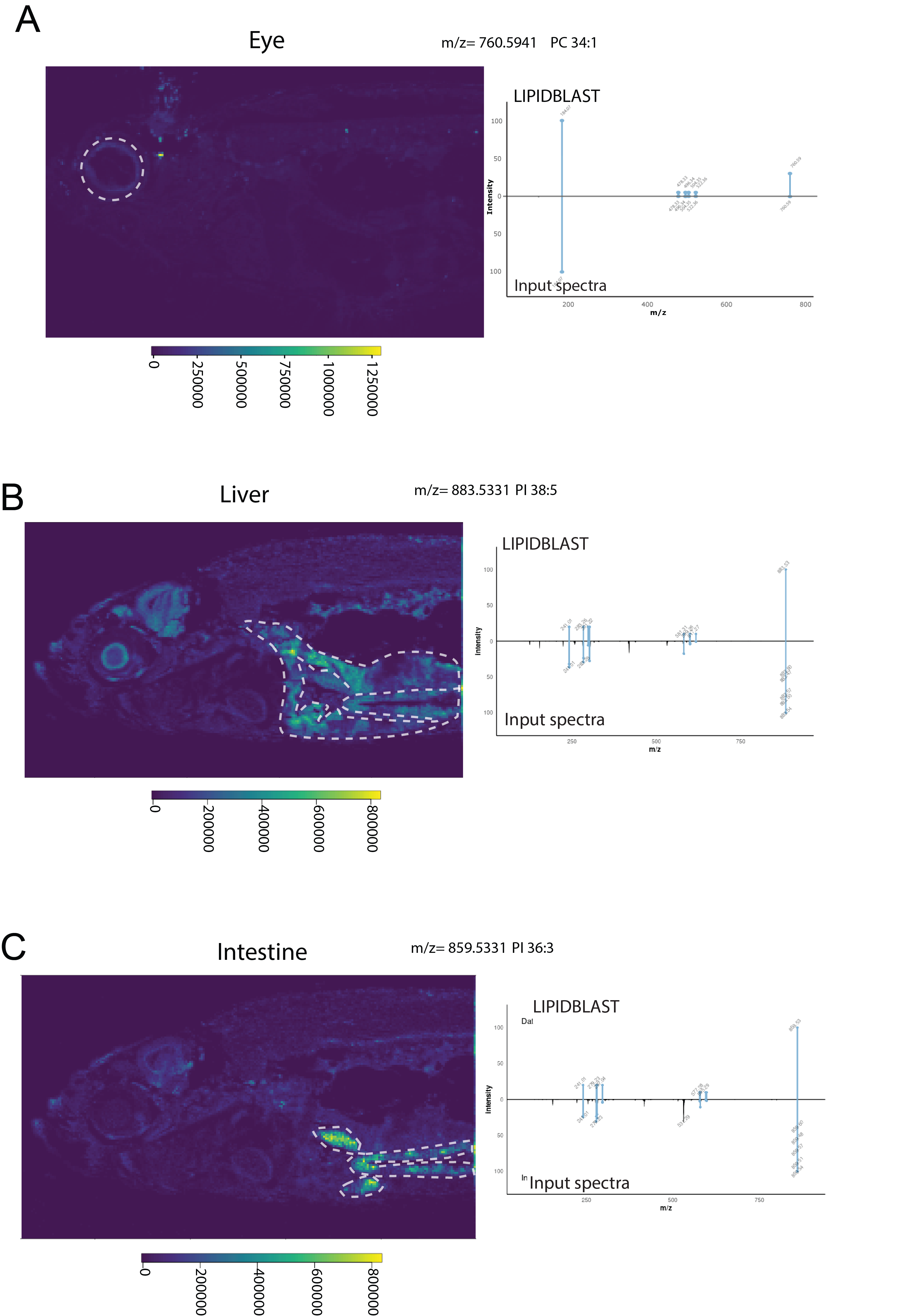

Supplement: vbag012_Supplementary_Data [file vbag012_supplementary_data.zip › Supplementary Fig 2.tif]

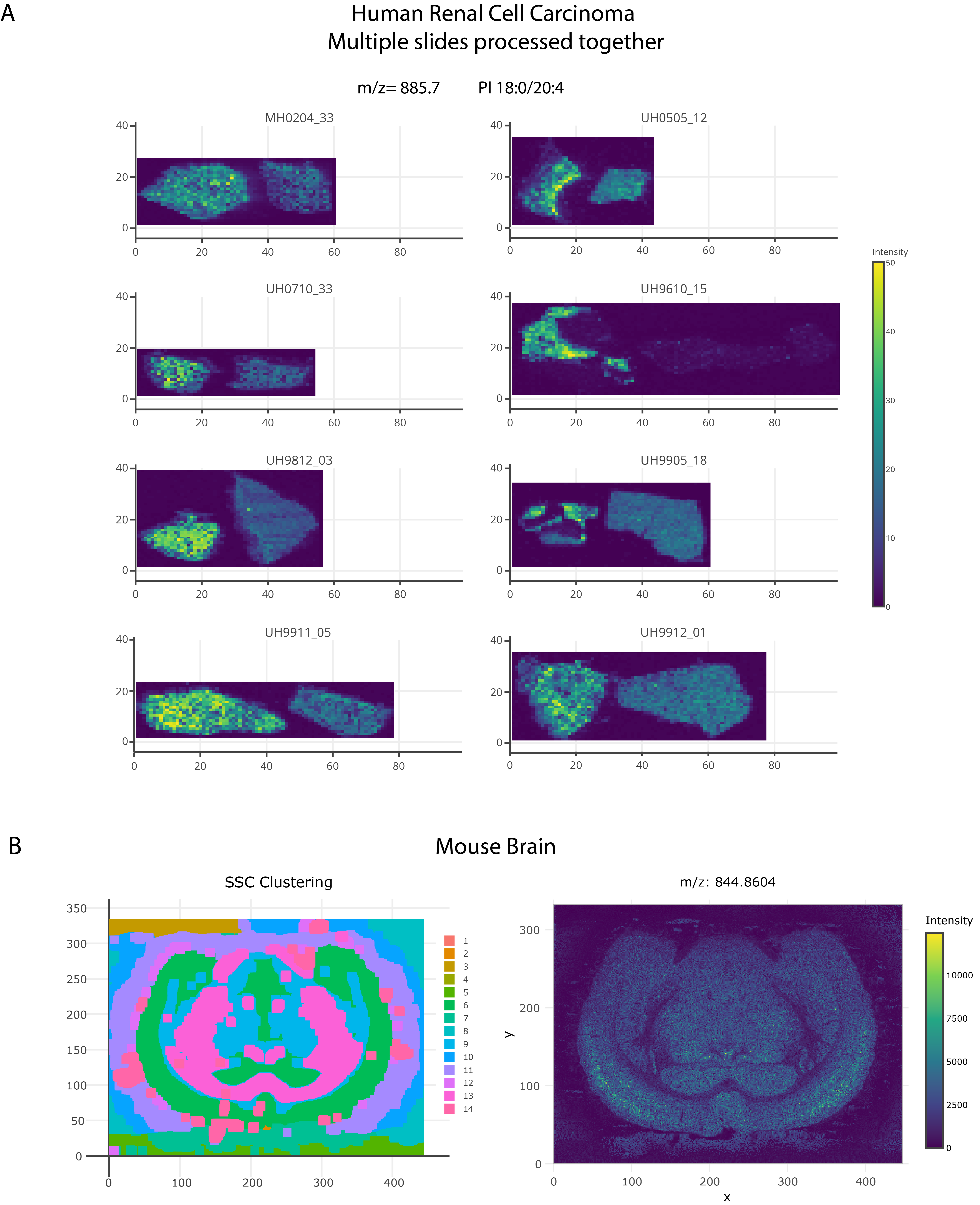

Supplement: vbag012_Supplementary_Data [file vbag012_supplementary_data.zip › Supplementary Fig 3.tif]
